# Learning to love ourselves again: Organizing Filipinx/a/o scholar-activists as antiracist public health praxis

**DOI:** 10.3389/fpubh.2022.958654

**Published:** 2022-08-19

**Authors:** Erin Manalo-Pedro, Andrea Mackey, Rachel A. Banawa, Neille John L. Apostol, Warren Aguiling, Arleah Aguilar, Carlos Irwin A. Oronce, Melanie D. Sabado-Liwag, Megan D. Yee, Roy Taggueg, Adrian M. Bacong, Ninez A. Ponce

**Affiliations:** ^1^Data and Research Committee, Filipinx/a/o Community Health Association, Los Angeles, CA, United States; ^2^Fielding School of Public Health, University of California, Los Angeles, Los Angeles, CA, United States; ^3^UCLA Center for Health Policy Research, Los Angeles, CA, United States; ^4^Division of General Pediatrics, Children's Hospital Los Angeles, Los Angeles, CA, United States; ^5^VA Advanced HSR Fellowship, Greater Los Angeles Healthcare System, Los Angeles, CA, United States; ^6^Department of Public Health, California State University, Los Angeles, Los Angeles, CA, United States; ^7^Medical College of Wisconsin, Milwaukee, WI, United States; ^8^Bulosan Center, University of California, Davis, Davis, CA, United States; ^9^Stanford University Center for Asian Health Research and Education, Palo Alto, CA, United States

**Keywords:** Pinayism, racism and antiracism, counterspaces, critical race theory, health equity, Filipino, Public Health Critical Race Praxis, community organizing & grassroots development

## Abstract

A critical component for health equity lies in the inclusion of structurally excluded voices, such as Filipina/x/o Americans (FilAms). Because filam invisibility is normalized, denaturalizing these conditions requires reimagining power relations regarding whose experiences are documented, whose perspectives are legitimized, and whose strategies are supported. in this community case study, we describe our efforts to organize a multidisciplinary, multigenerational, community-driven collaboration for FilAm community wellness. Catalyzed by the disproportionate burden of deaths among FilAm healthcare workers at the onset of the COVID-19 pandemic and the accompanying silence from mainstream public health leaders, we formed the Filipinx/a/o Community Health Association (FilCHA). FilCHA is a counterspace where students, faculty, clinicians, and community leaders across the nation could collectively organize to resist our erasure. By building a virtual, intellectual community that centers our voices, FilCHA shifts power through partnerships in which people who directly experience the conditions that cause inequities have leadership roles and avenues to share their perspectives. We used Pinayism to guide our study of FilCHA, not just for the current crisis State-side, but through a multigenerational, transnational understanding of what knowledges have been taken from us and our ancestors. By naming our collective pain, building a counterspace for love of the community, and generating reflections for our communities, we work toward shared liberation. Harnessing the collective power of researchers as truth seekers and organizers as community builders in affirming spaces for holistic community wellbeing is love in action. This moment demands that we explicitly name love as essential to antiracist public health praxis.

## Introduction

“They say some stories need to wait to be toldBut I need a way to unfoldAll of the weight of the people before meWho never got chances to speak from they soul”–Ruby Ibarra, “Skies”

A critical component for health equity lies in the inclusion of structurally excluded voices, such as Filipinx/a/o Americans^1^ (FilAms) (1). Because FilAm invisibility is normalized, denaturalizing these conditions rxequires reimagining power relations regarding whose experiences are documented, whose perspectives are legitimized, and whose strategies are supported (2–4). To spark this reimagination, we begin with a reminder from Pinay rapper Ruby Ibarra of the multigenerational burden generated from the silencing of FilAm stories.

In this community case study, we describe our efforts to organize a multidisciplinary, multigenerational, community-driven collaboration for FilAm community health. Catalyzed by the disproportionate burden of deaths among FilAm healthcare workers at the onset of the COVID-19 pandemic and the accompanying silence from mainstream public health leaders, we formed the Filipinx/a/o Community Health Association (FilCHA). FilCHA is a national public health organization dedicated to advancing health equity, justice, and wellbeing in the Filipinx/a/o community through community-driven, collaborative, and data-informed approaches.

FilCHA operates as a counterspace where students, faculty, clinicians, and community leaders collectively organize to resist our erasure, abolish oppressive structures, and promote our wellness. Wellness is the harmonizing of mind, body, emotion and spirit that connects self and community actualization (5, 6). By building a virtual, intellectual community that centers our voices, FilCHA shifts power toward people who directly experience the conditions that cause inequities have leadership roles and avenues to share their perspectives (7). We demonstrate the need for counterspaces as progressive values evolve in public health (8).

## Background and rationale

Current conceptions of antiracist public health praxis may not fully account for FilAm complexities. Partially due to racialization as Asian American model minorities, FilAms are infrequently included in structural racism research (9). Population-level analyses often invisibilize FilAms by neglecting to systematically collect racial/ethnic data and by aggregating Asian American subgroups (10). Upon disaggregation, traditional health indicators (e.g., high English proficiency, baccalaureate degree attainment, household income) do not correlate with positive health outcomes for FilAms who are more likely hypertensive, diagnosed with cancer, and disproportionately represented in healthcare COVID-19 deaths (1). More nuanced analyses that contextualize FilAm health are needed.

One such infrequently examined area in health research is FilAms' historical trauma, which stems from a turbulent colonial history with the United States (US) under the guise of benevolent assimilation (1, 11). The physical harm caused by the Philippine-American war resulted in the deaths of an estimated 20,000 Pilipino combatants and more than 200,000 civilians from combat and disease. As a means of cultural dispossession, generations of Pilipinos were then educated in a system “designed [for] the economic and political reality of American conquest” (12). The centrality of the US in early Pilipino migration and its evolution into a sophisticated system of federally-backed policies (e.g., the creation of the Overseas Employment Development Board in 1974) contribute to the continued economic reliance of the Philippines to export skilled labor out of the country (13), producing a global diaspora of displaced Pilipino families. As a survival strategy, FilAm families emphasized assimilation and acculturation. Across the US, even along the West and East coasts where most FilAms reside, FilAms may struggle to find community (2). Disrupting the multigenerational effects of this hegemonic miseducation and isolation requires reflection and action (14).

### Anti-racist public health praxis

Praxis refers to the constant cycle of reflection and action with an underlying commitment to naming and transforming the world (14). This iterative approach is reflected in the National Campaign Against Racism which names racism, asks how it operates, and organizes and strategizes to dismantle it (15). Public Health Critical Race Praxis, an antiracist methodology for public health research and practice, focuses on contemporary patterns of racial relations, knowledge production, conceptualization and measurement, and action (16). Although each focus has shaped our health equity work, we center our case study on knowledge production, given the paucity of public health literature on FilAm knowledge producers.

### Knowledge production

Scholars of color face barriers to knowledge production in traditional academic arenas which are rooted in colonialism, dehumanization, imperialism, unfreedom, and isolation (3, 17, 18). Amidst rigorous academic and professional programs, students of color are frequently exhausted by the barrage of overt and covert reminders of their otherness (19). They often taken on the burden of remediating these harmful racial climates through uncompensated labor (e.g., diversity committees, student organizing) (20). Similarly, Black, Indigenous, Latine,^2^ and Asian American faculty have enumerated their own microaggressions, connecting these everyday assaults to poor health outcomes and the denial of tenure (21, 22). As such, racial campus climates have been detrimental for the FilAm faculty pipeline (23–25).

In addition to devaluing bodies of color, white supremacy also denigrates the knowledges of communities of color. One inconspicuous yet powerful form of epistemic oppression is epistemicide, which refers to the “killing of knowledge systems” through the erasure of non-Western ways of knowing (26). The historical theft and cooptation of non-Western knowledge systems has implications for today's prospective knowledge makers (3, 27). That is, when students of color are deprived from knowing “how autonomous, resourceful, and abundant their civilizations were prior to their relationship to colonialism… it becomes difficult to imagine a world beyond colonial domination” (28).

Yet imagination can illuminate alternative paths toward health equity (7). People who have been, historically and presently, structurally excluded from societal privilege possess the unique ontological and epistemic privilege to know, most intimately, the conditions of their oppression (29). Notwithstanding the increased attention to community-partnered participatory research as an approach to centering community voices in public health research (30), dismantling the dominant narratives that dehumanize FilAms “requires that the people experiencing/embodying realities of oppression retain narrative control—not the credentialed” (7). That is, the margins of society serve as a site of possibility and resistance for inhabitants (31)—not a brief stopover for “health equity tourists” who seek to co-opt recent streams of resources intended to address racial injustice (32). Indeed, health equity scholar Lisa Bowleg has argued for critical epistemologies as necessary to dismantle harmful narratives and radically change the underlying conditions that structure exclusion (33, 34).

How do alternate knowledges advance antiracist public health praxis? In this case study, we examine how the Filipina American feminist lens of Pinayism can explain the development of FilCHA by centering FilAm narratives.

## Description of the case and methodological aspects

### Action: Critical race counterspaces

We were compelled to create FilCHA as a counterspace because existing academic public health and governmental institutions had not sufficiently prioritized FilAm health on a national level in general, and COVID-19 disparities in particular. Counterspaces emerged as a concept at the intersection of critical race theory and education (35). Initially theorized as a response to negative racial campus climates, counterspaces provide a safe community for marginalized people to bring their full selves to collectively engage in meaning-making and transformative change (36, 37). Whether as formally enrolled students, curious researchers, or lifelong learners, we who identify as members of marginalized communities seek “spaces that would support [our] ideas, [our] research, and [our] commitments” (38).

Counterspaces offer respite from microaggressions, deficit framing, and erasure through affirming relationship building (35, 36). Socorro Morales, a Critical Race Feminista, defines counterspace as an intellectual place for transformation where marginalized people bring their full selves in their complexities to critically discuss their overlapping and distinct realities (37). That is, counterspaces disrupt the invisibility and isolation that people of color face in academic settings, akin to the pedagogy of collegiality through which students “[learn] about community organizing while actually being a community” (39). Stretching beyond the confines of a single course, however, counterspaces also resemble an academic home, inviting multiple generations of interdisciplinary scholars and community members to vulnerably share raw ideas, critically yet caringly offer feedback, and generate novel insights rooted in the common struggle for justice (38).

Thus, one strategy for advancing health equity is to create counterspaces that facilitate knowledge production and promote actions toward holistic wellness. Producing knowledge in counterspaces enables us to reflect on our own epistemologies, analyze and critique oppressive systems that threaten our wellness, and act toward liberation (15, 40).

### Reflection: Intersectionality *via* Pinayism

To guide our inquiry, we turned to Pinayism, which offers a unique, intersectional standpoint on the dynamic inclusion/exclusion of FilAm voices. Originating in the 1920's, “Pinay” refers to the political identity of Filipinas in the United States (41). Ethnic studies professor Allyson Tintiangco-Cubales (42) conceptualized Pinayism as feminist praxis, connecting Pinay stories at the global, local, and personal levels to reveal the complexities of intersectionality for transformative change. Just as feminism is not limited to females, Pinayism is not limited to Pinays. Rather, Pinayism centers the experiences of Pinays to generate a deeper understanding of local issues by framing oppression globally and facilitating individuals' capacity to affect change (42). Thus, Pinayism aligns with recent calls to address contemporary public health crises by engaging in intersectional praxis (43).

As an individual and communal process of humanization, Pinayism offers a valuable perspective for deepening antiracist public health praxis. According to the decolonialist body of Sikolohiyang Pilipino (i.e., Filipino Psychology), colonialism ruptured kapwa, the Pilipino core value of unity with others, indicating a deep connection with and commitment to community (44–46). Pinayist scholars restore kapwa through a shared understanding of contemporary struggles through decolonial, feminist, and antiracist contexts (40). Critical kapwa pedagogy, a strategy for teaching FilAm students, builds on kapwa value as a pertinent approach for collective healing through humanization, wholeness, and decolonizing epistemologies (46). Other examples of scholarship related to community health include the colonial roots of chronic diseases in the FilAm community juxtaposed against the FilAm healthcare workforce (47); ethnic studies as community responsive wellness (5, 6); and antiracist mothering amidst the coronavirus pandemic (48).

Public health literature has yet to robustly engage Pinayism. Notably, scholar-activists Maglalang et al. (49) recently argued for infusing Asian American Studies into public health training to develop critical consciousness among future health educators and to enhance their capacity to address anti-Asian racism through practice. For example, Asian American Studies and feminist theory guided an empirical paper and revealed that family and economic considerations kept nurses in specific settings that increased COVID-19 exposure risk (50). However, explicit application of Pinayism to explain FilAm health inequities has yet to find its place within public health's peer-reviewed journals.^3^

Even when informed by historical context, health disparities scholarship often frames communities of color by their deficits (33, 51). In contrast, Pinayism aligns more closely with desire-based research (2), “intent on depathologizing the experiences of dispossessed and disenfranchised communities” (52). To generate a more complete story, Pinayism encourages self-determination through the individual and communal process of acknowledging pain, showing love, and engaging in reflection toward community actualization and liberation, as summarized by the equation, pain + love + reflection = liberation (40).

While initially articulating Pinayism as “pain + love = growth,” Tintiangco-Cubales also described it as a fluid concept focused on revolutionary action, self-affirming conduct, and a self-determining system (42). In keeping with valuing the multiplicity of Pinay experiences and the fluidity of self-determination, the elements of the equation remain unbound by rigid definitions to be imposed onto others. Rather, the intention is for Pinayists to engage in “deep self-reflection as a means toward collective liberation” (i.e., pain + love + reflection = liberation) (40).

As Pinay scholar-activists engaged with FilAm studies as a means for healing (46), the first and second authors proposed applying the lens of Pinayism to reflect on our involvement with FilCHA. We leveraged the Pinayism equation as an analytical tool for decomposing the elements that contributed to FilCHA as a liberatory space. Conscious of the tendency for FilAms to avoid conflict rooted in the deeply valued concept of pakikisama (i.e., getting along with others) (44), we chose to name our pain. Furthermore, to counter the primarily positivist and deficit-oriented field of public health, we also reflected upon our actions as love for our community. Lastly, to acknowledge the sociopolitical implications of historical trauma on contemporary struggles for health equity (53), we reflected on our humanizing practices in the context of the ongoing struggle for collective liberation.

For the Pinay “to name her scars… and to uncover its connections to her subjectivity” (54), both data and analytical lenses are grounded in Pinay experiences (48). We embrace the Critical Race Feminista concept of cultural intuition, which leverages both shared experiences and community memory to enhance our interpretation of data (55). Indeed positivist claims to objectivity are fundamentally inappropriate for feminist and intersectional epistemological analyses which posit that knowledge is socially constructed (33). Instead, rigor is evaluated by the logic used to link data to the stated propositions and addressing rival explanations for findings. Case studies on unusual cases emphasize departures from convention to reveal insights about the status quo (56). Similarly, examining the proposition that FilCHA serves as a counterspace reveals how mainstream systems of knowledge production in public health have hindered FilAm health research.

We drew on two forms of data collection to make sense of FilCHA's development. We reviewed documentation (e.g., emails, agendas, internal documents, social media) that chronicled our milestones. Concurrently, we reflected together on our process as active members and/or co-founders, and thus participant-observers, to facilitate our meaning-making of what transpired. The authorship group for this commentary includes five members of the inaugural FilCHA board, including senior researchers and current students. In fact, the majority of authors are graduate students or recent alumni from programs in public health, medicine, and sociology. Although we all identify as FilAms, each author's lens has been shaped by lived experiences in various regions of the US, tied to different familial contexts of migration.

## Results

We summarize our findings through the Pinayism process of pain + love + reflection = liberation.

### Pain: Invisibility reinforces inaction

The Pain experienced by the FilAm community during the pandemic is multifaceted and rooted in a long history of colonial violence dating back to the 16th century under Spanish rule. Since the late 1500's, Spain, and subsequently the US from 1899 to 1946, exploited Philippine society, land, and people, uprooting connections to place, history, nature, spirit, ancestry, and community through various tools of colonialism, imperialism, and racism (57). As a result of this historical trauma, FilAms experience psychological and social hardships that ultimately manifest as health problems (1). Yet in pursuit of higher education to address these health problems, FilAm students often experience unrecognized marginalization, isolation from other FilAm students and mentors, siloed scholarship on FilAm issues which contribute to disciplinary ignorance, and institutional inaction to remedy said challenges (58). Ecologically, these harms further marginalization across individual, interpersonal, community, and societal levels. In Table 1, we highlight a non-exhaustive list of interdisciplinary publications that historicize examples of Pain experienced by the FilAm community. Omitting this context, which otherwise would inform why knowledge production remains a challenge, further perpetuates the epistemicide that thwarts attempts to alleviate the Pain.

**Table 1:** Manifestations of pain experienced by the Filipinx/a/o American community by ecological level.

**Ecological level *Pain***	**Further interdisciplinary readings on manifestations of *Pain***
Individual: Invisibility	Not being seen in America (59, 60) Exclusion from curriculum (61, 62) Few political leaders (63)
Interpersonal: Isolation	Difficulty finding other FilAms due to underrepresentation in academia (23, 25, 58)
Community: Ignorance	Epistemicide (i.e., “killing of knowledge systems”) in education (2), psychology (64), and public health (1)
Society: Inaction	Inaction on a structural level to address above issues through policy (63), the health care workforce (65), and clinical research (66)

This reading list is not intended to be exhaustive.

The pandemic exacerbated the ongoing disinvestment in FilAm health on a national and global scale. Early on, we witnessed a disproportionate number of deaths among FilAm healthcare workers. Despite FilAms comprising 4% of registered nurses, they made up nearly 30% of COVID-related deaths, according to the National Nurses United, a statistic underscored by the poignant digital memorial KANLUNGAN, which was curated by the transnational anti-imperialist feminist organization, AF3IRM.^4^ Against the backdrop of America's racial reckoning, anti-Asian hate crimes, and global exploitation of Filipino health care workers, the pandemic compounded the burdens of Pinay racial equity scholars (48). Acknowledging the multilayered pain inflicted on the FilAm community serves as the first step to adequately address the continued marginalization we have historically endured.

### Love: Collaboration *via* counterspace

**Figure 1:**
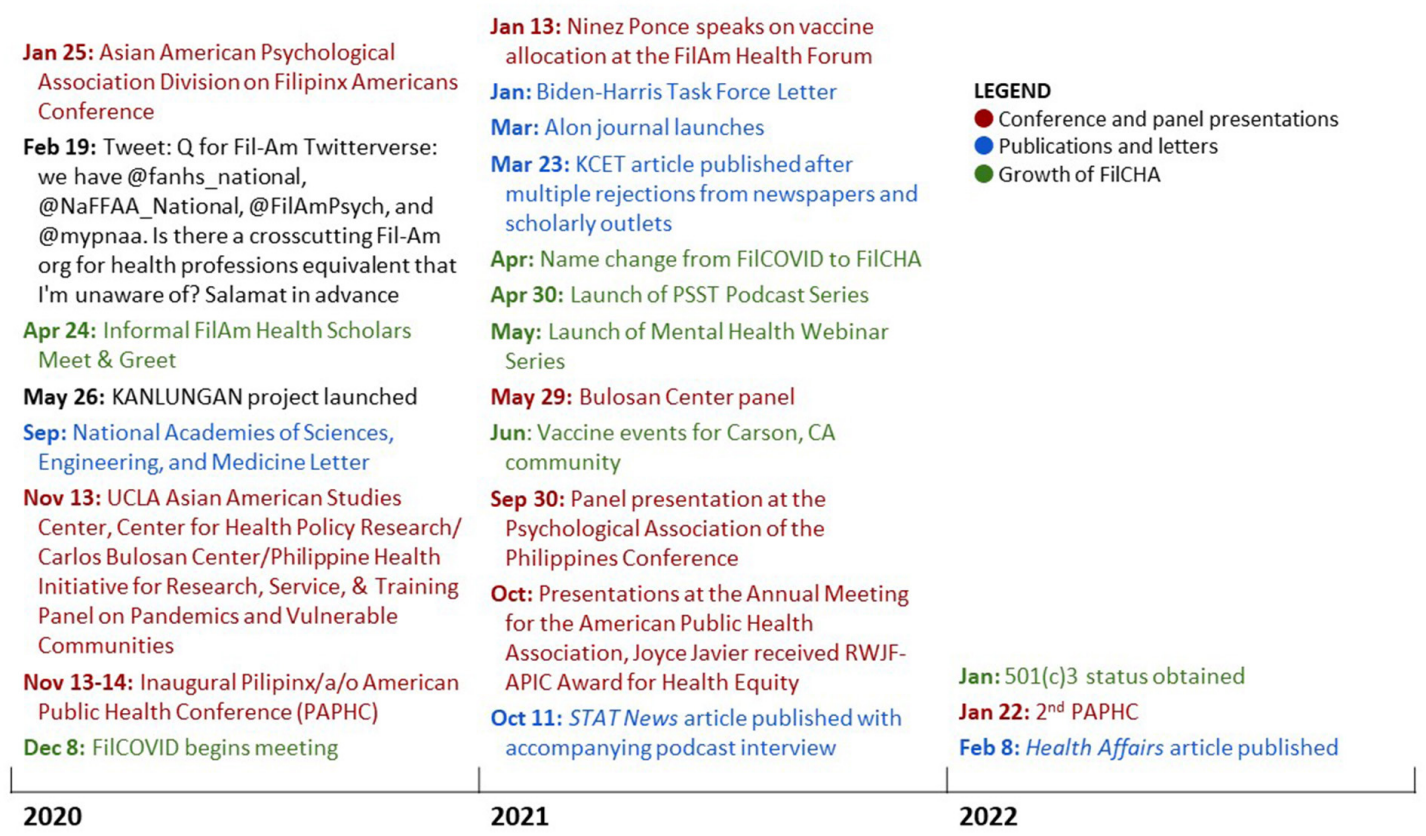
Timeline of the development of the Filipinx/a/o Community Health Association, 2020–2022.

FilCHA's origins preceded the pandemic. Drawing upon a wealth of shared community resources [e.g., aspirational, resistant, navigational, social forms of community cultural wealth (67)], FilAms previously established local grassroots organizations and professional associations in medicine, nursing, psychology, education, and ethnic studies to reimagine ways to heal the bodies, minds, and souls of our community (40). In public health, however, there remained a void that pan-Asian organizations had not filled (68). Longing for an interprofessional space that centered our epistemologies in the discourse, we organized toward our collective liberation and healing in public health, as illustrated in the timeline in Figure 1 (40).

As the COVID-19 pandemic accelerated over the early months, high-profile media articles calling attention to the burden on FilAm nurses^5^ brought urgency and prompted FilAm public health scholars to connect and organize virtually. A shared recognition of the invisibility of FilAm COVID-19 disparities in cases and deaths culminated with a September 2020 letter to the National Academies of Sciences, Engineering, and Medicine. Addressing the COVID-19 Vaccine Allocation Framework, the letter served as an act of resistance by critiquing the context of ongoing FilAm health inequities. In December 2020, signatories of the letter gathered to form an *ad hoc* group, the Filipinx/a/o COVID-19 Resource and Response Team (FilCOVID), largely inspired by the National Pacific Islander COVID-19 Response Team (69). Initially, we sought to educate and recommend policies for FilAm COVID-19 equity, yet our mission transformed into engaging in decolonization, self-determination, humanization, and relationship building within public health.

**Figure 2:**
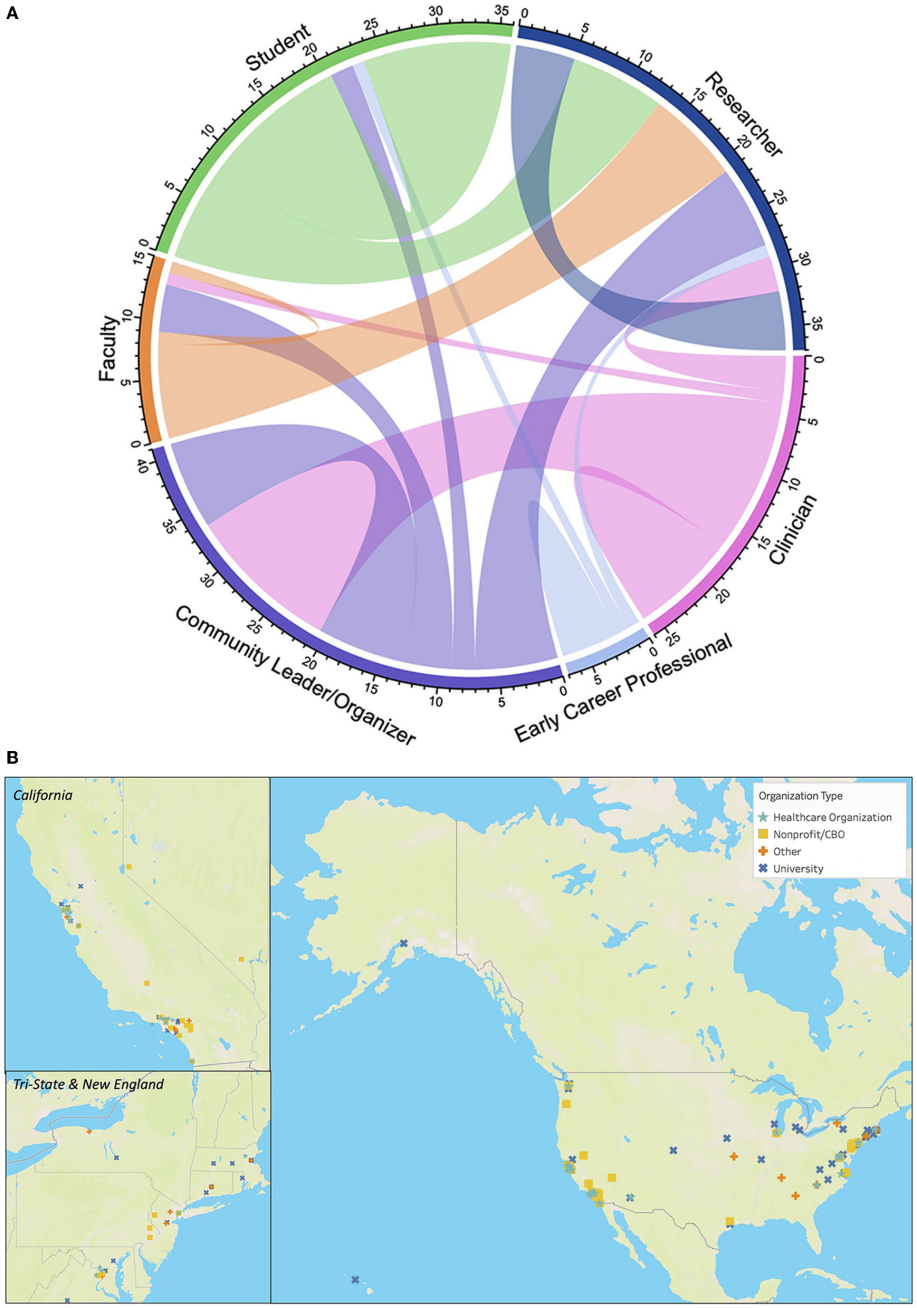
Diversity and geographic reach of the Filipinx/a/o Community Health Association network. **(A)** Composition of Filipinx/a/o Community Health Association membership illustrating diversity and overlapping of roles. Chords that connect to the same role indicate members who serve one role. Chords that connect from one role to another represent members who serve in both roles. Clinician is inclusive of community-based direct service providers. Generated with circlize package in R (70). **(B)** Geographic distribution of organizations from more than 600 individuals who signed the January 2021 policy letter to the Biden-Harris Health Equity Task Force regarding COVID-19 and the Filipinx/a/o American community.

Through weekly Zoom meetings, inviting colleagues from other networks, and a growing social media presence, FilCOVID rapidly grew in size and influence. FilCOVID evolved into FilCHA, a transdisciplinary, interprofessional, and intergenerational organization consisting of over 100 members. Our members have diverse academic trainings and backgrounds in ethnic studies, history, sociology, anthropology, political science, epidemiology, policy, economics, social work, psychology, health services research, community health sciences, and medicine. FilCHA members include students, community leaders and organizers, researchers, clinicians and community-based direct service providers, faculty, and early career professionals, with many of our members serving in multiple roles. Figure 2A illustrates and quantifies the diversity in the roles of our members: out of 36 students, 8 are also researchers; out of 26 clinicians, 13 are also community leaders; out of 12 faculty members, 4 are also community leaders and organizers. As a virtual, intellectual space, FilCHA has become a hub for FilAm health equity scholar-activists where our Filipinx/a/o communities' epistemologies can be centered in our discussions and organizing.

The influential impact and broad reach of our members enabled us to compile more than 600 signatures within a few days for a January 2021 policy letter to the Biden-Harris Health Equity Task Force regarding COVID-19 and the FilAm community.^6^ Illustrating the geographic spread of the signatories, Figure 2B displays the geographic location of the signatories' organization affiliations. The overwhelming response across multiple sectors (e.g., non-profit, academic, professional) provided us the momentum and validation needed to organize for health equity. The swift mobilization of our community emerged because of the combination of love, care, commitment, knowledge, responsibility, respect, and trust (71). The letter reached across different networks, sharing a recognition of pain but the power of the counternarrative. In a time of crisis, the letter offered context and space to understand, challenge, and transform established belief systems (72–74). Further, the letter provided new possibilities and connections for those on the margins.

Recognizing that COVID illustrated the broader issue of FilAm invisibility in public health discourse and practice, members clarified its mission. FilCOVID transitioned to the Filipinx/a/o Community Health Association (FilCHA), a collective non-profit space we created to honor our ancestors, share hope and joy, and individually and collectively heal by identifying holistic wellness through self-determination. We adopted a broader vision to ensure visibility, accurate data, and equitable allocation of resources for the Filipinx/a/o community, empowering new and existing movements to create sustainable healing.

By combining our interprofessional, multidisciplinary membership with a digital presence, FilCHA inhabits a unique space in the FilAm community. We are not directly affiliated with a specific academic institution. Rather, we pair the strengths of academic perspectives with a community focus and partner with other community-based organizations. This allows us to coordinate and build kapwa with each other nationally and outside of academic institutions that have not traditionally valued the importance of critical cultural production of engaged scholarship that expresses our own perspectives in public health. Benefiting from elders' expertise with incorporation, we formalized the national organization by acquiring 501(c)3 non-profit designation in January 2022.

### Reflection: Producing knowledge

To overcome the erasure of FilAm knowledge in health equity discourse, we collectively redefined, restored and redistributed power by gathering and analyzing our own knowledge to raise visibility within the field of public health and the broader FilAm community. Table 2 lists knowledge produced by FilCHA members: policy letters, publications, presentations, and podcast episodes. Upon seeing the continued omission of FilAms from COVID-19 equity discussions, we collaboratively drafted policy letters in hopes of addressing structural change in the areas of vaccine allocation, data collection and disaggregation, and classification as underrepresented minorities, including our letter to the Biden-Harris Health Equity Task Force.

**Table 2:** Knowledge produced by Filipinx/a/o Community Health Association members, December 2020–May 2022.

**Policy letters**
1. Letter to the National Academies of Sciences, Engineering, and Medicine (September 2020)
2. Letter to the Biden-Harris Health Equity Task Force (January 2021)
**Publications**
1. A rapid assessment of the impact of COVID-19 on Asian Americans: cross-sectional survey study
2. Access and utilization of mental health services among Pacific Islanders.
3. Addressing the interlocking impact of colonialism and racism on Filipinx/a/o American health inequities
4. Asian, Latinx, or Multiracial? Assessing Filipinxs' Health Conditions and Outcomes by Aggregate Ethnic Category
5. Between Women of Color: The New Social Organization of Reproductive Labor
6. Capturing Racial/Ethnic Diversity in Population-Based Surveys: Data Disaggregation of Health Data for Asian American, Native Hawaiian, and Pacific Islanders (AANHPIs)
7. Characterizing Awareness of Pre-Exposure Prophylaxis for HIV Prevention in Manila and Cebu, Philippines: Web-Based Survey of Filipino Cisgender Men Who Have Sex With Men
8. Characterizing Problematic Drug Use Among Transgender Women and Cisgender Men During the Emerging HIV Crisis in the Philippines: Implications for Policy Research
9. Colonial mentality and psychological flexibility among Filipinx Americans
10. Condom Use and Social Capital Among Filipinx Transgender Women and Cisgender Men Who Have Sex with Men (Trans-WSM and Cis-MSM): a Structural Equation Modeling
11. Considerations of Racism and Data Equity Among Asian Americans, Native Hawaiians, and Pacific Islanders in the Context of COVID-19.
12. Creating a Culture of Mental Health in Filipino Immigrant Communities through Community Partnerships
13. Creating a shared definition of adolescent mental health in the Filipino American community: A comparative focus group analysis.
14. Deferred depression? Mediation analysis of Deferred Action for Childhood Arrivals and immigration enforcement among Undocumented Asian and Pacific Islander students
15. Disaggregation for Health Equity: Shedding Light on COVID-19's Impact on the Filipinx Community
16. Doctoral Students as Agents for Change: Shaping Our Public Health Training Environment
17. Evaluating an Evidence-Based Parenting Intervention Among Filipino Parents: Protocol for a Pilot Randomized Controlled Trial.
18. Evaluating Translation of HIV-Related Legal Protections Into Practice: A Qualitative Assessment Among HIV-Positive Gay, Bisexual, and Other Men Who Have Sex With Men in Manila, Philippines
19. Executive summary of the 2020 clinical practice guidelines for the management of hypertension in the Philippines
20. Filipinx American Studies: Reckoning, Reclamation, Transformation
21. Frank Mancao's “Pinoy Image”: Photography, Masculinity, and Respectability in Depression-Era California
22. From imperialism to inpatient care: Work differences of Filipino and White registered nurses in the United States and implications for COVID-19 through an intersectional lens
23. Health disparities for Filipinxs in health care are disguised by data aggregation
24. Health Equity and Enrollment in Preventive Parenting Programs: A Qualitative Study of Filipino Parents
25. Heterogeneity in migrant health selection: The role of immigrant visas
26. Heterogeneity in the association of citizenship status on self-rated health among Asians in California
27. Internalized transphobia and mental health among transgender adults: A nationwide cross-sectional survey in South Korea
28. Intimate partner violence and HIV testing in Filipino women: analysis of the 2017 Philippine National Demographic and Health Survey
29. Life Satisfaction and Social and Emotional Support Among Asian American Older Adults.
30. Lost on the frontline, and lost in the data: COVID-19 deaths among Filipinx healthcare workers in the United States
31. Recasting the immigrant health paradox through intersections of legal status and race
32. Responsive Medical Providers and Recent HIV Medical Services Engagement Among Transgender Women and Cisgender Men Who Have Sex With Men in the Philippines
33. The Association Between Moderate and Serious Mental Health Distress and General Health Services Utilization Among Chinese, Filipino, Japanese, Korean, and Vietnamese Adults in California.
34. The COVID-19 pandemic and pediatric mental health: advocating for improved access and recognition
35. The Impact of Structural Inequities on Older Asian Americans During COVID-19
36. Transgender-specific developmental milestones and associated experiences of violence, discrimination, and stigma among Filipinx transgender women who are sexually active with men
37. Transpacific Rizalistas: Portrait Photography and the Filipino Becoming-Subject
38. US Health Care Relies on Filipinxs While Ignoring Their Health Needs: Disguised Disparities and the COVID-19 Pandemic
39. Using structural equation modeling to characterize multilevel socioecological predictors and mediators of condom use among transgender women and cisgender men who have sex with men in the Philippines
**Presentations, panels, webinars**
1. A Filipinx physician on the health disparities disguised by data (Stat News First Opinion Podcast)
2. Enduring Impact of Colonialism on Health Inequities in the US (A Health Podyssey)
3. Examine the relationships between Fil Am identity, COVID-19 stressors, and mental health outcomes
4. How can CHW address disparities and management of DM, HTN, and asthma in the Asian community (APHA 2021)
5. Lost in the Frontlines, Lost in the Data, and Lost in the Policy Priorities (Filipino American Health Forum on COVID-19)
6. “Missing” context, “missing” data: An ecosocial assessment of the disproportionate COVID-19 burden among filipina/x/os (APHA 2021)
7. No Health without Mental Health
8. Path to Public Health Panel Presentation at the 2022 Pilipinx American Public Health Conference
9. Pinay Perspectives on the Lakas Mentorship Program: Reflecting on our Power to Heal, Care, and Resist (2021 Bulosan Center for Filipinx Studies Research Conference)
10. Power of Positive Parenting in Filipino Families: Helping Our Youth Thrive During COVID-19 (Philippine Embassy)
11. Presentation at the 2021 Council of Young Filipinx Americans in Medicine Conference
12. Promoting multidisciplinary and international collaboration to address mental health (2021 Psychological Association of the Philippines Conference)
13. Public Health Research and Our Community: The Filipinx/a/o Covid-19 Response Team Research and Data-Disaggregation Subcommittee (2021 Bulosan Center for Filipinx Studies Research Conference)
14. Racism & Health Virtual Symposium (Health Affairs Briefing)
15. Reclaiming the full spectrum of Filipinx/a/o sexual and reproductive health care (2021 Bulosan Center for Filipinx Studies Research Conference)
16. Stop Asian Hate: The Mental Health Impact of Racial Discrimination Among Asian Pacific Islander Young and Emerging Adults during COVID-19 (AcademyHealth Annual Research Meeting 2021)
17. Webinar series on self-care and wellbeing
18. White House Initiative on Asian Americans, Native Hawaiians, and Pacific Islanders Virtual Celebration for Filipino American History Month 2021
**Podcast episodes**
1. Pilot episode
2. COVID-19 vaccine and general information
3. Asian American Hate Crimes Effects on the Filipinx/a/o Community
4. Understanding of Mental Health: Self-Care and Community Care during COVID-19
5. Filipinx/a/o Healthcare Workers COVID-19 Experiences
6. COVID-19 Impacts on the Filipinx/a/o LGBTQAI+ Community
7. Importance of Data, Data Disaggregation, and Research during COVID-19
8. Addressing COVID-19 Misinformation in the Filipinx/a/o Community
9. Parenting, Family Dynamics, and School Roll-Out during COVID-19
10. COVID-19 Effects on Filipinx/a/o Adolescents and Young Adults
11. COVID-19 Effects on Filipinx/a/o Biracial and Multiracial Community
12. Road to Recovery in All Forms during COVID-19
13. Immigration and Resources for Mixed Status/Undocumented Filipinx/a/o during COVID-19
14. Community Organizing during COVID-19 (West Coast/Midwest/East Coast)

Podcast episodes can be accessed at: https://www.filcha.org/podcast-archives.

In facilitating research on and with FilAms, FilCHA members published at least 39 articles since December 2020. Many of the publications emphasized the health and social needs of FilAms. Our multidisciplinary collaboration led to an article on colonialism and racism on FilAm health inequities in the *Health Affairs* special issue on racism and health (1). The infrequent integration of sociological and historical context, chronic disease, mental health, and policy relevance generated new research questions for future studies with the potential to tangibly improve FilAm health. Additionally, we developed community resources that have been fundamental for disseminating FilAm knowledge and strengthening future FilAm power relations: a publicly viewable bibliography of FilAm health articles and an email distribution list for members to share funding opportunities, policy updates, community-defined healing services, and study participant recruitment. An online repository for FilAm oral histories and other datasets is under development.

Leveraging extensive networks built over decades of organizing, we also engaged the broader FilAm community to cocreate knowledge through a funded podcast series, webinars, and presentations. We preserved experiences of FilAms at various intersections of oppression while highlighting strategies for healing. FilCHA decentered positivist approaches to research by uplifting kuwentuhan (talking story) through our 14-episode podcast series, titled “The Pilipinx/a/o Sharing Stories Together (PSST) Podcast.”^7^ PSST alludes to the sound that FilAms often make to get someone's attention. The podcast featured perspectives from clinicians, researchers, community organizers, creatives, youth, parents, undocumented immigrants, LGBTQI+, and multiracial FilAms. The podcast gained traction on social media platforms^8^; on Twitter, FilCHA's largest social media platform, 191 tweets about the podcast amassed over 204,600 impressions. Similarly, on Facebook 99 posts about the podcast reached 16,437 individuals. Our mental health webinars incorporated activities such as karaoke, cooking, and Zumba, reaching more than 2,000 individuals. Through more than a dozen presentations, we shared our ideas with diverse audiences including public health professionals, FilAm youth, and mental health professionals in the Philippines.

These collective successes were built upon the intellectual and emotional labor of prior rejections as individuals. One of the earliest collaborations started in May 2020. After connecting on Twitter, two FilCHA co-founders decided to draft an op-ed article on data disaggregation for COVID-19 data, centering their argument on the growing number of FilAm healthcare worker deaths. Days later, AF3IRM launched the KANLUNGAN tribute website and the UCLA Center for Health Policy Research launched the COVID-19 Race/Ethnicity and Risk Factors Dashboard. Enhanced with new data sources and a senior third author, the op-ed was submitted. Month after month, while the deaths of FilAm healthcare workers kept climbing, news outlets and scholarly journals rejected the op-ed. Facilitated through the senior author's media recognition, the article finally found its home accompanying KCET's Power & Health documentary^9^ in March 2021.

While this persistence may be admirable, it is also exhausting. In various committee meetings, several FilCHA members expressed similar frustrations over rejected publications, grant applications, and panel presentations that centered on FilAms. An established FilAm researcher shared that grant reviewers commented that studies that focused only on FilAms were not warranted. Rather than processing these setbacks individually, FilCHA created a space for being vulnerable, exposing common struggles, and sustaining each other despite setbacks. This unrecognized labor takes a toll. Similarly burdened, counterspaces have “nurtured what would become a growing lineage of U.S. Filipino education scholar-activists” (38). We extend this idea toward growing a lineage of U.S. FilAm scholar-activists for health equity.

### Liberation: Decolonization, humanization, self-determination, and relationship-building

Through FilCHA as a counterspace, we have built pathways toward collective healing. Transforming conditions to reach health equity requires continuous collective commitment to liberation from all oppression. Indeed, as a community-driven effort, sustaining counterspaces depends on individual and communal praxis. FilCHA engages in decolonization, humanization, self-determination, and relationship-building practices (40).

Acknowledging the legacies of colonialism on FilAm health within public health literature is just the first step toward decolonization. FilCHA seeks to advance self-determination and healing with other peoples who continue to be impacted by colonial legacies. For example, our contribution to the *Health Affairs* special issue on racism and health has sparked cross-racial conversations regarding anticolonialism (1). Alongside American Indian/Alaska Native, Native Hawaiian and Pacific Islander, and other Asian American scholars, our members have advocated for visibility through disaggregated data (10, 75, 76). This current paper attempts to bridge ethnic studies, critical race theory in education, and public health praxis. To transform public health, we continue to invest in cultivating health social movements, learning critical theories, decolonizing methods, and health promoting practices from antiracist scholars and community organizers across disciplines.

Driven by our shared humanity, we prioritize telling the stories of FilAms within and beyond FilCHA. The podcast was one of several ways we brought attention to the nuances of FilAm experiences (e.g., multiracial, undocumented, queer). We also spotlighted FilCHA members on social media platforms, highlighting reasons for joining, relevant projects, and experience with the COVID-19 vaccine. Additionally, in the scholarship we produced, we often referenced the KANLUNGAN tribute website to the more than 200 healthcare workers of Philippine descent who passed away from COVID-19. All of this is intentionally in contrast to the dominant epidemiological representations of people of color who are often presented as statistics devoid of context (77). As members of the FilAm community, we embody our ancestors' resistance and struggle, sharing experiences with those whose health outcomes we prioritize.

Moreover, FilCHA facilitates our self-determination by setting our own agenda and mobilizing our vast network to increase our impact. We share the common goal to serve, address, and answer FilAm community health and wellbeing needs. Covering the areas of research, policy, outreach, and mental health, FilCHA committees provide the infrastructure for supporting member-driven initiatives. Current collaborations include this manuscript, participation in the #WeCanDoThis campaign, online “Zoomba” workshop for wellbeing, administratively advocating for equitable health polices at the federal level, and intentionally mentoring rising public health leaders.

Finally, the relational culture within FilCHA signifies the ongoing reclamation of kapwa. In contrast to elitism and individualism, we welcome and support members at whichever stage they are in their journey. Decisions are consensus-driven, rather than defaulting to those with seniority or professional roles conventionally seen as most prestigious (e.g., physician). Though many members are credentialed, respect and familiarity are extended through the honorifics of “Ate,” “Tita,” or “Ninang” (older sister, auntie, or godmother) rather than “Doctor.” Having survived marginalization in their own careers, elders share their wisdom, networks, and resources. Opportunities for skill development (e.g., speaking at conferences, coauthoring manuscripts, and leading projects) are shared during virtual meetings and through online collaboration tools (e.g., Google Groups, Slack, and social media). Challenging dominant values that breed competition, FilCHA seeks to reunite our fellow FilAms by seeing ourselves in each other.

## Discussion

“There is power in seeing each other. …in hearing each other. …in speaking to each other. There is power in ‘us.' There is power because they cannot fathom what we can do together.” –Cee Carter and Korina M. Jocson (78).

The development of FilCHA demonstrates how community-driven efforts disrupt epistemicide, an overlooked expression of structural racism against Asian Americans. Through disciplinary self-critique, our case study advances public health praxis by articulating the need for counterspaces to facilitate self-determined knowledge production. Further, by leveraging Pinayism as our analytical tool, we exhibited how epistemic privilege aids our collective understanding of the complex barriers to health equity. We encourage a sincere love for the people toward a more humanizing public health.

As people affected by the legacy of colonialism, we, as public health scholar-activists, sought to recover our significance and “learn to love ourselves again” (79, 80). By naming our collective pain, building a counterspace for love of the community, and generating reflections for our communities, we work toward shared liberation. Analyzing the development of FilCHA through the lens of Pinayism guided our approach to understand our actions, not just in the current crisis State-side, but through a multigenerational, transnational understanding of what knowledges have been taken from us and our ancestors (27). Digital artifacts, such as meeting minutes, generated within counterspaces can help to illuminate differences from the normalized processes of knowledge production. Additionally, participant observation embraces reflexivity, acknowledges the situatedness of the researcher within the research, and promotes cultural intuition (55, 81). Counterspaces illuminate the possibilities for antiracist public health praxis by placing people of color at the center of knowledge production and case study as a method offers another tool for understanding the complexity of racism and antiracist public health praxis (56).

The racialization of FilAms as colonized “model minorities” without unmet health needs sparked the development of FilCHA. As an example of preparation meeting opportunity, the coronavirus pandemic catalyzed a critical mass of FilAm scholars and community leaders to engage in online conversations around the disproportionate burden among the FilAm community and their roots in colonial legacies. This ignited our agency and creativity to not only make meaning of our community's unequal exposure to COVID-19 but also comprehend the limited response from conventional public health organizations.

Exclusion from both mainstream public health efforts and conventional “minority health” initiatives to curb COVID-19 spurred us to act. FilCHA was birthed by connecting existing networks of FilAm community organizations and Asian American public health entities through our shared struggle to achieve holistic community wellbeing. In broader discussions of racial health equity, attention to FilAm health tends to waver between the omission altogether of Asian Americans from population health analyses, positioning as white-adjacent model minorities to denigrate other communities of color, and, less frequently, inclusion within disaggregated Asian American data (1). Indeed, education scholars have argued that the incessant need for FilAms to develop counterspaces is perpetuated by insidious, systemic underfunding and inequitable decision-making structures (2).

Liberation fundamentally requires an ongoing commitment to transform the spaces we occupy (82). Abolishing oppressive systems must be motivated by emotion (83). Love is enacted by sustaining each other and sharing the collective responsibility (71, 84). Efforts to address anti-Asian hate should also seek to cultivate love.

### Contradictions

As a globally displaced diaspora, FilAms are undeniably complicit as settlers on stolen land (85). As we prioritize consciousness raising to highlight FilAm stories of resistance and struggle, how FilCHA relates to Indigenous people from the Philippines has yet to be determined.^10^ As the organization and its' membership grow, FilCHA aims to intentionally wrestle with the incommensurability of FilAm health and Indigenous sovereignty (85).

Through our ongoing process of (un)learning, we benefit from the intellectual work advanced by Indigenous scholar-activists [e.g., decolonizing methodologies (3), historical trauma (53, 86, 87), loss of culture (88), colonial schooling (89)]. We encourage anticolonial stances in public health literature in hopes that increased attention to colonialism as a determinant of health will yield more collective power and resources to disrupt it.

As a precursor to unsettling stolen land, we assert that we must learn to love ourselves, our people, and our culture, to cultivate the desire and means to reunite with our homeland. Notably, the exodus continues; 46,000 Pilipinos immigrated to the U.S. in 2018 (90). By engaging in self-determined antiracist praxis, we aim to make visible the mechanisms through which white supremacy and U.S. imperialism have and continue to maintain the conditions that separate FilAms from our wholeness and our potential for collective liberation.

### Implications

Our case study advances the idea that antiracist public health praxis is both an individual and a collective process facilitated through counterspaces. This cannot be done in isolation and fundamentally contradicts public health's long held focus on individuals as the unit of analysis and the assumed neutrality of researchers. In solidarity with other communities that struggle for visibility, we offer lessons learned and recommendations for shifting attention to the margins through kapwa (Table 3).

**Table 3:** Lessons learned and recommendations for promoting kapwa.

**Lesson learned**	**What happened with FilCHA**	**Recommendations**
**Find each other**. Humanization necessitates the destruction of the hierarchies and categories that separate people from recognizing each other's sacred humanity.	Our inner kapwa led us to find each other and build power in community toward liberation. This also facilitated a flattened hierarchy and consensus-driven decision-making. The stay-at-home orders pushed community organizers to pivot to online spaces, increasing visibility beyond the local community and access despite geographic spread. Through well-connected members, we came together as a network. Trusted colleagues helped to identify funding opportunities that facilitated crucial operational support and provided honoraria for podcast guests from the community. Motivated by kapwa, we sought to uplift what already existed, not to compete.	Identify community cultural wealth within own networks. Identify the responsibility, relevance, and relationships with community. State and federal organizations, foundations, and local health departments should increase the pipeline of structurally excluded communities into academic and research careers. State and federal organizations, foundations, and local health departments should be evaluated on the funding opportunities they provide to match the diverse geographic, racially/ethnically, LGBTQ+, needs of their service area. Use disaggregated race/ethnicity data to inform state, federal, and local policies and research funding priorities. Invest in developing best practices guides, conferences, evaluations, and pedagogical tools for starting, growing, and maintaining a network.
**Bring our full selves**. Embracing our wholeness requires vulnerability, acknowledging struggles, and being receptive to love.	We learned to unapologetically embrace our Filipino-ness as scholars, including how it shapes our understanding of public health and healing. As members of the community we sought to serve, we offered each other grace as we each took time to step back when needed. Honestly sharing the struggles we were going through allowed workloads to be redistributed, facilitated collaborative learning opportunities, and attained achievements beyond our individual imagination. As an unstaffed organization, members are drawn to volunteer because of love for the community. However, from a practical sense, funding has enabled us to provide leaders with modest project-based stipends.	Commit to transformation of health and wellbeing through humanization, hope, and healing. Utilize Ethnic Studies Pedagogies and principles in public health to develop critical consciousness and agency. Foundations, local health departments, state and federal organizations should provide funding opportunities and technical assistance for organizations, services, and interventions rooted in and guided by love and community centered healing to start, grow, and maintain a network promoting health equity.
**Tell our stories**. Decolonizing epistemologies means activating our agency to produce the knowledge that will bring our community closer to liberation.	FilCHA became a space for exposing struggle, shifting the way we understand power, power relations, and how we have struggled with them. We created a space where we were free to explore and choose our own beliefs and actions to address our community wellness and wholeness. Free from self-doubt, microaggressions, and having to justify why FilAms deserve to be understood, our creativity flourished, producing knowledge for our people. Despite our shared commitment to the FilAm community, the extensive diversity of FilCHA's membership inevitably generates different perspectives. We occasionally navigate difficult discussions through which we challenge each other.	Produce art, movement, performance, projects, businesses, and scholarship that expresses structurally excluded perspectives, counternarratives, medicine, and knowledge. Provide public access to disaggregated data. Foundations, local health departments, state and federal organizations should invest funds into community stories, community defined practices, networks, and languages to drive health education, research, services, and policy. Create opportunities for self-determination that enable community members' intersectional epistemologies to guide decision-making.

FilAm, Filipinx/a/o American; FilCHA, Filipinx/a/o Community Health Association. Kapwa refers to the Pilipino core value of unity with others, indicating a deep connection with and commitment to community.

We must interrogate how population-level approaches erase the experiences of structurally excluded groups, including FilAms. We push back on the notion that FilAm health is only worth studying or intervening upon when juxtaposed against whiteness (2). We commit to exposing the structures that reject grant proposals and scientific papers on the faulty logic that rationalize FilAms as undeserving of investment or attention. Rather, examining, enacting, and advocating for FilAm health is warranted because of our complexities and inherent deservingness. Multidisciplinary, community-led organizing for public health rooted in love for the people is essential for disrupting our continued erasure and promoting community wellness.

FilAms and other overlooked communities would benefit from institutionalized investment in interdisciplinary collaboration and self-determination. This paper briefly touched on a few strengths-based approaches [e.g., desire-based research (52)] and community-based healing [e.g., critical kapwa (46) and community responsive wellness (5)]. Numerous other ways of knowing exist. Scholarship on anti-Asian racism and public health could benefit from theories rooted in ethnic studies (91), critical race theory (92), and decolonizing methodologies (2).

The conditions surrounding FilCHA were unique but developing counterspaces for antiracist public health praxis is not only feasible but imperative for health equity. “Resistance should not be mistaken for remedy” (2) but rather an opportunity to engage in reflection on how public health must transform. Regardless of numeric representation, tackling health equity remains elusive if lived experiences of those students and community members are not genuinely valued as legitimate ways of knowing (33). Though not formally theorized as a counterspace, the National Cancer Institute's pre-doctoral Minority Training Program in Cancer Control Research has exemplified a humanizing space for graduate students of color to engage in collective meaning-making since 1999 (93). We recommend that health workforce diversity programs similarly prioritize the humanization of scholars of color and encourage public health educators to promote concepts from critical race theory of education (e.g., counterspace) and intersectional epistemologies (e.g., Pinayism) (7, 94).

## Conclusion

Pinayism guided our realization that kapwa has been our strategy for survival. Using critical kapwa for our collective healing restores our shared power, driven not by obligation but a loving commitment to community (46). FilCHA emerged out of an interconnected network of relationships. As with deep systems changes that originate from small interactions and relationship building, it was through the tweets, letters, and conversations that built FilCHA as a counterspace to humanize, heal, and decolonize. FilCHA has become a site for exposing struggle and reclaiming collective power.

The field of public health is not immune to the white supremacist culture and structures upon which the United States was founded. Dismantling anti-Asian racism requires more than documenting incidence rates of overt hate. Harnessing the collective power of researchers as truth seekers and organizers as community builders in affirming spaces for holistic community wellbeing is love in action. This moment demands that we explicitly name love as essential to antiracist public health praxis.

## Data availability statement

The raw data supporting the conclusions of this article will be made available by the authors, without undue reservation.

## Author contributions

All authors listed have made a substantial, direct, and intellectual contribution to the work and approved it for publication.

## Funding

This work was supported by the Pilipinx/a/o Sharing Stories Together (PSST) Podcast series would not have occurred without the Association of Asian Pacific Community Health Organizations, whose administrative support and prior success with the Koviki Talk Podcast for Pacific Islanders led to a grant through the Walmart Foundation. Open access publication fees were provided by the UCLA Center for Health Policy Research. RB reports support from the Agency for Healthcare Research and Quality as part of the Los Angeles Area Health Services Research Training Program (T32 Predoctoral Fellowship) during the conduct of this study. CO reports support from the Veterans Affairs Office of Academic Affiliations through the VA Advanced Health Services Research Fellowship, during the conduct of the study.

## Conflict of interest

The authors declare that the research was conducted in the absence of any commercial or financial relationships that could be construed as a potential conflict of interest.

## Publisher's note

All claims expressed in this article are solely those of the authors and do not necessarily represent those of their affiliated organizations, or those of the publisher, the editors and the reviewers. Any product that may be evaluated in this article, or claim that may be made by its manufacturer, is not guaranteed or endorsed by the publisher.

## Author disclaimer

The contents do not represent the views of the authors' employer or institution, including those of the US Department of Veteran Affairs and the US government.

## Footnotes

^1^We use “Pilipino” to refer to people with ancestral ties to the regions of Luzon, Visayas, and Mindanao residing in what is now known as the Republic of the Philippines. We use the term “Filipinx/a/o American” as a gender-inclusive term for people with Philippine ancestry residing in what is now known as the United States of America. We also incorporate “Pinay” as a political reference to the inseparability of U.S. imperialism on Filipinas in the United States.

^2^We use “Latine” to refer to people with ancestral ties to the region now known as Latin America. Latine was introduced by queer, non-binary, and feminist Spanish speakers to push back against gender and heteronormative bias (https://callmelatine.com/2020/12/14/an-open-letter-to-allies/).

^3^PubMed search for “Pinayism” produced 0 results. “Pinay” yielded 2 results that were not related to the author last name “Pinay”: one was a 2010 article on action research in reference to a Filipino Canadian domestic worker organization and the other was a 2021 reflection from a Pinay social worker that did not explicitly reference “Pinayism.”

^4^ https://www.kanlungan.net/

^5^https://www.statnews.com/2020/04/28/coronavirus-taking-outsized-toll-on-filipino-american-nurses/, https://www.latimes.com/california/story/2020-07-21/filipino-americans-dying-covid, https://www.nbcnews.com/news/asian-america/largest-share-migrant-nurses-entire-u-s-filipino-community-hit-n1237327

^6^ http://tiny.cc/filcovidletter

^7^ https://www.filcha.org/podcast-archives

^8^https://twitter.com/filxao_cha, https://instagram.com/filxao_cha, https://www.facebook.com/FilxaoCHA

^9^ https://www.kcet.org/shows/power-health/disaggregation-for-health-equity-shedding-light-on-covid-19s-impact-on-the-filipinx-community

^10^ https://www.ikatvoices.com/
